# Dual Drug Delivery of Sorafenib and Doxorubicin from PLGA and PEG-PLGA Polymeric Nanoparticles

**DOI:** 10.3390/polym10080895

**Published:** 2018-08-09

**Authors:** György Babos, Emese Biró, Mónika Meiczinger, Tivadar Feczkó

**Affiliations:** 1 Institute of Materials and Environmental Chemistry, Research Centre for Natural Sciences, Hungarian Academy of Sciences, Magyar tudósok körútja 2., H-1117 Budapest, Hungary; babos@mukki.richem.hu (G.B.); biro@mukki.richem.hu (E.B.); 2 Research Institute of Biomolecular and Chemical Engineering, University of Pannonia, Egyetem u. 10, H-8200 Veszprém, Hungary; meiczinger@mukki.richem.hu

**Keywords:** sorafenib, doxorubicin, polymeric nanoparticles, drug delivery

## Abstract

Combinatorial drug delivery is a way of advanced cancer treatment that at present represents a challenge for researchers. Here, we report the efficient entrapment of two clinically used single-agent drugs, doxorubicin and sorafenib, against hepatocellular carcinoma. Biocompatible and biodegradable polymeric nanoparticles provide a promising approach for controlled drug release. In this study, doxorubicin and sorafenib with completely different chemical characteristics were simultaneously entrapped by the same polymeric carrier, namely poly(d,l-lactide-*co*-glycolide) (PLGA) and polyethylene glycol-poly(d,l-lactide-*co*-glycolide) (PEG-PLGA), respectively, using the double emulsion solvent evaporation method. The typical mean diameters of the nanopharmaceuticals were 142 and 177 nm, respectively. The PLGA and PEG-PLGA polymers encapsulated doxorubicin with efficiencies of 52% and 69%, respectively, while these values for sorafenib were 55% and 88%, respectively. Sustained drug delivery under biorelevant conditions was found for doxorubicin, while sorafenib was released quickly from the PLGA-doxorubicin-sorafenib and PEG-PLGA-doxorubicin-sorafenib nanotherapeutics.

## 1. Introduction

Hepatocellular carcinoma (HCC) is one of the most destructive cancers. At present, sorafenib is the only drug available that prolongs the life of patients with HCC. However, non-specific uptake leads to high toxicity and serious side effects. Sorafenib is a multikinase inhibitor that targets various receptor tyrosine kinases and RAF kinases; hence, it hampers tumor growth and exerts cytostatic effects and thus demonstrates a significant overall survival rate of patients, e.g., with HCC. However, its water immiscibility results in low bioavailability [1]; thus, a high dosage is required. Doxorubicin is a common chemotherapeutic agent in numerous cancer therapies [2]. It is an anthracycline antibiotic. Doxorubicin hydrochloride salt is a water-soluble, hygroscopic, crystalline form of the drug, which possesses better bioavailability. Doxorubicin activation on the nucleic acids of dividing cells can occur by intercalation between the base pairs of the DNA strands, thus inhibiting the synthesis of DNA and RNA by impeding the replication and transcription in the cells and producing iron-mediated free radicals that destroy cell membranes, proteins, and DNA. The most disadvantageous side effects of doxorubicin are myelosuppression and cardiotoxicity.

The drawbacks of the use of anticancer agents could be decreased by the application of a nanocarrier that supports the targeted drug delivery and controls the release of effective agents. Polymeric nanoparticulate drug delivery systems have been shown to be a valid approach to sustain drug liberation and to enable a targeting function. There are some existing papers on sorafenib or doxorubicin microencapsulation using PLGA copolymers. Nevertheless, the sorafenib loading in PLGA nanoparticles is generally rather low. E.g., a 1.4% sorafenib loading in PLGA nanoparticles with an oil-in-water single-emulsion solvent evaporation method was achieved in [3]. Multiblock polymer nanoparticles consisting of (poly(lactic acid)-poly(ethylene glycol)-poly(l-lysine)-diethylenetriamine pentaacetic acid and the pH-sensitive material poly(l-histidine)-poly(ethylene glycol)-biotin could encapsulate 2.4% sorafenib [4]; however, by a nanoprecipitation-dialysis method using a block copolymer of dextran and poly(d,l-lactide-*co*-glycolide) the realized drug content was substantially higher, with a maximum of 5.3% [5]. Doxorubicin-loaded PEG-PLGA-Au nanoparticles with a cytostatic drug content of 3.9% were prepared to enable combined treatment based on chemotherapy and heat-therapy by near-infrared radiation in [6].

By simultaneous delivery of anticancer drugs to tumor cells, a synergistic effect can be realized by an appropriate composition [7]. In some studies, co-delivery of sorafenib and doxorubicin has already been successfully done. E.g., a nanocomposite composed of doxorubicin containing a polyvinyl alcohol core and a human serum albumin-sorafenib shell was manufactured by a sequential freeze-thaw method followed by ethanol coacervation in [8]. The drug loading and the encapsulation efficiency of doxorubicin were 3.0% and 82.0%, respectively, in the nanocore; these values for sorafenib were 2.4% and 91%, respectively, in the albumin nanoshell. Lipid-polymer hybrid nanoparticles decorated with the tumor-homing peptide iRGD were prepared in [9]. The hybrid nanocomposites possessed synergistic cytotoxicity, a pro-apoptotic ability, and improved uptake by HepG2 human hepatocellular carcinoma cells. The blood circulation time and bioavailability and antitumor effects were also significantly increased in HCC xenograft mouse models. Although the drug loading for sorafenib was rather low (3.6%), high doxorubicin content (13.6%) was realized in this work. Very recently, Xiong et al. [10] entrapped sorafenib adamantine-terminated doxorubicin using poly(ethylene glycol)-β-cyclodextrin. Their reduction-responsive supramolecular nanosystem was manufactured through host-guest interaction between cyclodextrin and adamantine moieties, which then self-assembled into regular spherical nanoparticles that showed an inhibitory effect against HepG2 hepatocellular carcinoma cells.

In our study, PLGA and PEG-PLGA carriers, respectively, were used to entrap doxorubicin and sorafenib together in nanotherapeutics in order to enable the anticancer drugs to exert a synergistic influence. The double emulsion solvent evaporation method was applied for the simultaneous entrapment of the drugs. After the optimization of size and encapsulation efficiency, the drug release profile was investigated in human blood plasma. In vitro cellular studies in HT-29 cancer cells were performed to study the cellular uptake and cytotoxicity of the drug-loaded nanocomposites.

## 2. Materials and Methods

### 2.1. Materials

Poly(d,l-lactide-*co*-glycolide) (PLGA) polymer, Resomer RG 752H (lactide:glycolide: 75:25, inherent viscosity 0.14–0.22 dL/g, *M*_w_ = 4000–15,000), and Resomer RG 502H (lactide:glycolide: 50:50, inherent viscosity 0.16–0.24 dL/g, *M*_w_ = 7000–17,000 g/mol) were produced by Boehringer Ingelheim (Ingelheim am Rhein, Germany). PEGylated-PLGA (PEG-PLGA) polymer, Resomer, RGP d 5055 (PEG-PLGA) (PEG content: 3–7% (m/m), inherent viscosity: 0.93 dL/g, *M*_w_ = 33,500 g/mol) was obtained from Evonik (Essen, Germany). Polyvinyl alcohol (PVA, *M*_w_ = 30,000–70,000 g/mol, 87–90% hydrolysed), dichloromethane (DCM), acetone, glacial acetic acid, dimethyl sulfoxide (DMSO), sodium azide, 1-ethyl-3(3-dimethylaminopropyl) carbodiimide (EDC), *N*-hydroxysuccinimide (NHS), sodium dodecyl sulphate (SDS), 3-(4,5-dimethylthiazol-2-yl)-2,5-diphenyltetrazolium bromide (MTT), piroxicam, and RPMI-1640 medium were obtained from Sigma Aldrich (St. Louis, MO, USA). Sorafenib (free base) and doxorubicin HCl were purchased from Active Biochem (Hong Kong, China). Cyanine 5 amine was produced by Lumiprobe GmBH (Hannover, Germany).

### 2.2. Preparation of Nanocomposites

For the preparation of our dual-agent nanocomposites, the water-in-oil-in-water double emulsion solvent evaporation process was found to be appropriate. Briefly, the inner water phase was composed of 0.2 mL 0.5% (*w*/*v*) doxorubicin HCl solution in MilliQ water, which was added to the organic phase that consisted of 20 mg encapsulating polymer (Resomer RG 752H, Resomer RG 502H, or Resomer RGP d5055) dissolved in 1.0 mL DCM combined with 1.0 mg sorafenib dissolved in 0.1 mL acetone. The first emulsification was performed by sonication using a sonicator (Sonics Vibra Cell VCX 130, 130 W, Newtown, CT, USA) at an amplitude of 30% for 30 s. Then, the prepared water-in-oil emulsion was pipetted into the outer water phase that consisted of 1% (*w*/*v*) PVA in 5 mL phosphate buffer (pH 8). The water-in-oil-in-water emulsion was formed by another sonication at an amplitude of 50% for 60 s. The organic solvents were evaporated by magnetic stirring for 3 h under atmospheric pressure at room temperature. Nanoparticles were centrifuged by a Hermle Z216 MK microcentrifuge (Schwerte, Germany) at 15,000 rpm for 20 min, washed thrice, and redispersed in MilliQ water or phosphate-buffered saline (PBS, pH 7.4).

### 2.3. Investigation of Nanocapsules

#### 2.3.1. Morphology and Size Analysis

The morphology of nanocapsules was monitored after centrifuging and redispersing them in distilled water, dropping them onto a grid, and drying them under room temperature. Then, they were examined with an FEI Apreo scanning electron microscope (SEM, Thermofisher, Waltham, MA, USA) at 20 kV.

The size distribution of the obtained nanoparticles was determined by a Zetasizer Nano ZS (Malvern Instruments, Malvern, UK) operated with dynamic light scattering. The particles were characterized by their intensity mean diameter and polydispersity index (PDI).

#### 2.3.2. Nanoparticle Yield and Encapsulation Efficiency

The yield of the nanocomposites was determined by gravimetry after washing and drying of a known volume of nanoparticle suspension. The drug loading and encapsulation efficiency were investigated directly by dissolving 10 mg nanoparticles in 1 mL DMSO, and the solution was diluted to be detectable in the linear calibration range (1–20 mg/L). The absorbance of the solutions was measured spectrophotometrically (PG Instruments T80, Leicestershire, UK) at the absorbance maxima of doxorubicin (480 nm) and sorafenib (270 nm) in DMSO. The encapsulation efficiency of the active agents was calculated as follows:

Encaps. efficiency (%) = (mass of drug in nanocomposite/mass of total loaded drug) × 100

#### 2.3.3. In Vitro Drug Release Experiment

The in vitro drug release of the nanocomposites was investigated in human blood plasma and in ammonium acetate buffer (pH 5.5) because of the acidic tumor microenvironment. For in vitro release experiments after the washing steps, a 2 mL suspension including 4.4 mg PLGA or 6.4 mg PEG-PLGA nanocomposites was resuspended in 15 mL human blood plasma containing 0.03% sodium azide bactericide. Five milliliters (5 mL) of nanoparticle suspension in the release medium were pipetted to 5 mL non-transparent Eppendorf tubes, incubated at 37 °C in a G24 Environmental Incubator Shaker (New Brunswick Scientific Co. Inc., Edison, NJ, USA), and shaken by a BIO RS-24 Mini-rotator (Biosan, Rīga, Latvia) for 7 days at 700 rpm. Three parallel samples per nanocomposite were investigated. After 1 h and every 24 h, 0.5 mL from each sample were centrifuged (Hermle Z216 MK microcentrifuge, Gosheim, Germany) for 20 min at 15,000 rpm, washed three times, and the pellet was dissolved in 0.5 mL DMSO.

The sorafenib and doxorubicin concentration was measured by a Young Lin YL 9100 HPLC instrument (YL Instruments Co., Ltd., Gyeonggi-do, Korea) at 30 °C. The active agents were separated by a Zorbax SB-Aq column (150 mm × 4.6 mm, 5 μm; Agilent, Santa Clara, CA, USA). The mobile phase composition is given in Table 1. The flow rate was adjusted to 1 mL/min. The detection wavelength of sorafenib and doxorubicin was 280 and 480 nm, respectively. Piroxicam was used as an internal standard during the measurements.

The concentration of sorafenib and doxorubicin was calculated using calibration curves and the encapsulation efficiencies were calculated as follows:

Encapsulation efficiency (%) = (mass of drug in NP/mass of total loaded drug) × 100

### 2.4. Attachment of Fluorescent Dye

A 1 mL nanoparticle suspension (2.2 mg/mL PLGA and 3.2 mg/mL PEG-PLGA) was centrifuged and washed with MilliQ water, redispersed in 0.5 mL PBS (pH 7.4), mixed with 0.1 mL PBS (pH 7.4) involving a 50× molar excess of EDC and NHS related to the (PEG-)PLGA concentration, then incubated for 60 min at 25 °C, centrifuged and washed with MilliQ water, and redispersed in 1.0 mL PBS (pH 7.4). The obtained carbodiimide-activated nanoparticle dispersion was pipetted to a 0.02 mL PBS (pH 7.4) solution containing 0.5 mg/mL Cyanine 5 amine fluorescent dye and incubated for 1 h at 25 °C. Then, the nanocomposite dispersion was centrifuged, washed three times, and redispersed in 1 mL PBS.

### 2.5. Cell Cultures

The human cancer cell line HT-29 was grown in RPMI-1640 medium supplemented with 10% fetal calf serum (FCS) and 100 U/mL penicillin. The cells were cultured at 37 °C in a humidified atmosphere containing 5% CO_2_. They were trypsinized, resuspended, and precultured before use.

### 2.6. In Vitro Cellular Uptake and Cytotoxicity Studies

The HT-29 cellular uptake of the nanoparticles was evaluated using flow cytometry. The cells were cultured in 24-well plates at a cell density of 2 × 10^5^ cells per well at 37 °C for 24 h. After cultivation, 100 mg of fluorescently labelled nanoparticles/well were added to the cells and incubated for 24 h. Cells grown without nanoparticles were used as a negative control. The cells were washed by PBS, trypsinized, and redispersed in PBS containing 2% BSA. Flow cytometry was performed on a BD FACSAria III Cell sorter (BD Biosciences, San Jose, CA, USA) at an Ex/Em wavelength of 633/660 nm. Every sample was analyzed in triplicate.

The in vitro cytotoxicity caused in HT-29 cells was assayed using MTT reagent. Cells were seeded (10,000 cells/well) in 96-well plates. After 24 h of pre-incubation, the growth media were replaced with 200 μL of fresh RPMI-1640 medium containing 10% FCS and PLGA- and PEG-PLGA blank nanoparticles or the dual-drug-entrapping nanocomposites. Three different doxorubicin concentration levels of the added nanopharmaceuticals were applied: 0.5, 1.0, and 2.5 μg per well. The nanocomposites also contained sorafenib; however, it was a higher amount of sorafenib (with 6% and 28% using PLGA and PEG-PLGA, respectively) since its entrapment was more efficacious. The positive control samples were also supplied with the same amount of free doxorubicin and sorafenib in DMSO solution. DMSO cytotoxicity (without drugs) was also investigated. After 48 h of incubation, 20 mL/well MTT solution (5 mg MTT/mL) and 0.2 mL/well supplemented culture media were added followed by further incubation for 2 h. The supernatant was removed, and MTT lysis solution (DMSO, 1% acetic acid, 10% SDS) was added into each well to dissolve the cells with MTT formazan crystals. The absorbance was determined at 492 nm by a Robonik Readwell Touch (Navi Mumbai, India) plate reader. The percentage of viable cells was calculated by comparing the absorbance of treated cells against that of the untreated cells (negative control). The data were presented as the mean and standard deviation with eight replicates.

## 3. Results and Discussion

### 3.1. Preliminary Method Development

Our aim was to synthesize nanocomposites that are capable of encapsulating doxorubicin and sorafenib with high loading, high yield, and high encapsulation efficiency as well as a small size. Though PLGA copolymers are very frequently applied nanocarriers, to our knowledge co-loaded sorafenib and doxorubicin PLGA and PEG-PLGA nanotherapeutics have not been prepared so far. This fact is not surprising, because the solubility of the two drug molecules differs substantially; hence, it is not an easy task to involve them in a matrix comprised of one polymer. As was shown in the literature survey, hybrid nanosystems have mostly been used for their co-entrapment.

It must be emphasized that the conditions described in the experimental section were selected after extensive process-optimizing experiments, which also included some trials of nanoprecipitation and single emulsion methods. Nevertheless, these procedures are not described here in detail, since it was proved early in these examinations that they were not suitable for the efficient co-encapsulation of our active agents. Very briefly, for nanoprecipitation, a joint solvent of the active agents and the encapsulating polymer would be necessary; thus, doxorubicin HCl was converted to a free base using trimethylamine [11] before the process. However, the desalted doxorubicin could not be completely dissolved by the applicable solvents (ethanol, acetone, THF); thus, the nanoprecipitation process did not result in desirable encapsulation. In the oil-in-water emulsion solvent evaporation probe, the desalting of doxorubicin HCl was done during the emulsification; however, the doxorubicin encapsulation efficiency was too low in this case.

Since the solubility of doxorubicin decreases with increasing pH, the double emulsion solvent evaporation technique can be a suitable tool to microencapsulate doxorubicin effectively using an outer water phase with a pH higher than 7 [12]. In this approach, doxorubicin is included in the inner water phase. Because sorafenib is an organic soluble drug, it could be entrapped by the polymers after dissolving it in the organic phase. The nanocomposites formed by the double emulsion solvent evaporation method are characterised in the following sections.

### 3.2. Size, Yield, Drug Encapsulation Efficiency, and Drug Encapsulation Content

Particles smaller than 10 nm are quickly cleared by the renal filtration [13], while the ones bigger than 300 nm can be easily recognized by the reticuloendothelial system (RES) and removed from the blood circulation [14]. Thus, nanoparticles ranging from 10 to 200 nm could extravasate from the disorganized tumor vasculature to the tumor microenvironment due to tumor angiogenesis. Therefore, the manufacture of nanoparticles less than 200 nm in size and with a negative surface charge are desirable to prevent protein adsorption and promote accumulation in tumors. From low molecular weight PLGAs, such as Resomer RG502H and Resomer RG752H, the typical available particle sizes range from 60 to 200 nm [15].

**Figure 1:**
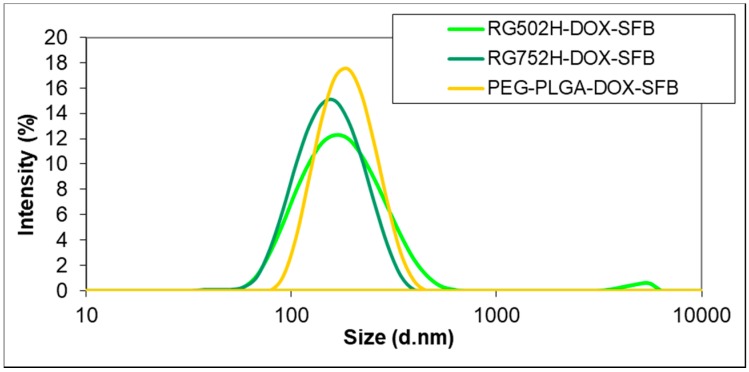
Size distribution by intensity of doxorubicin (DOX)- and sorafenib (SOR)-containing PLGA (Resomer RG502H and Resomer RG752H) and PEG-PLGA nanoparticles.

Although the Resomer RG 502H PLGA provided substantially higher encapsulation efficiencies than the Resomer RG 752H PLGA for both of the drugs, the size (164.6 nm) and PDI (0.203) of their nanocomposites were significantly larger (Table 2). The relatively high PDI indicates the presence of bigger particles formed by the separate precipitation of the drug during the solvent’s evaporation from the polymer nanoparticles, which is also supported by the size distributions (Figure 1). Such high PDI values were also found in our preliminary nanoprecipitation experiments or, e.g., by Lin et al. [16], who prepared PLGA-sorafenib nanocomposites with nanoprecipitation (PDI 0.21–0.35).

**Figure 2:**
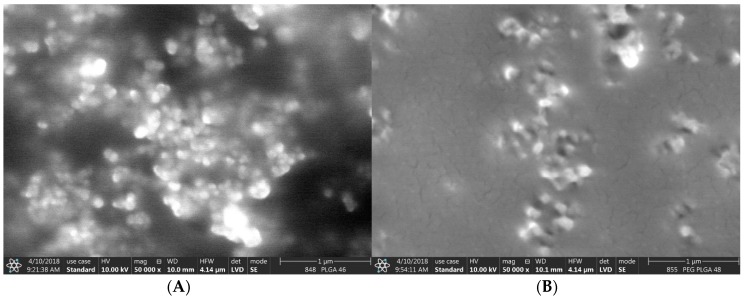
Scanning electron microscopic images of doxorubicin- and sorafenib-containing PLGA (**A**) and PEG-PLGA (**B**) nanocomposites.

SEM images of the nanocomposites (Figure 2) suggested significantly smaller nanoparticles than found by Dynamic Light Scattering (DLS) measurements (Figure 1). This can be interpreted as the fact that the DLS method displays the hydrodynamic diameter of the nanocomposites while SEM shows them in a dry state. We cannot exclude that the aggregation of some smaller particles occurred, which can also indicate a higher size during the DLS study. This latter hypothesis might be supported by the zeta potential measurements, which provided relatively low negative values, which means that their aggregation might have taken place. Neither of them showed variation as a function of encapsulating polymer, but varied in the narrow range between −17.6 mV (PLGA) and −18.8 mV (PEG-PLGA). Since the PVA surfactant cannot be completely removed from the surface of the nanoparticles due to its strong adsorption, they are sterically stabilized, which cannot be characterized by zeta potential measurements. This may be the main reason why a difference between the zeta potential values of the two types of nanocomposites was not found.

**Figure 3:**
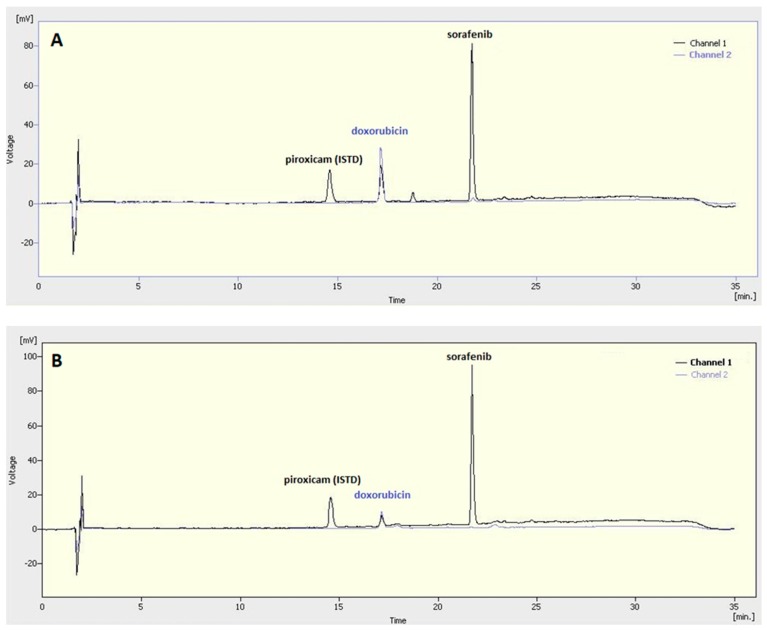
HPLC plot of dissolved sorafenib- and doxorubicin-loaded PLGA (**A**) and PEG-PLGA (**B**) nanocomposites.

Doxorubicin has absorbance in the visible range; thus, its concentration could be measured by UV–Vis spectrophotometry. Sorafenib absorbs exclusively in the UV region, while the absorbance of doxorubicin in the same UV region is also considerable although substantially lower than that of sorafenib. Because of their overlapping in the UV region, the HPLC method was applied to determine the concentration of both active agents after the dissolution of the nanocomposites. Sorafenib concentration was measured at a wavelength of 280 nm (Channel 1, Figure 3), while doxorubicin was analysed at 480 nm (Channel 2, Figure 3).

The highest yield (75.3%) and sorafenib encapsulation efficiency (88%) were achieved by the PEG-PLGA polymer (Table 2). Its doxorubicin encapsulation efficiency is also satisfactory (69%). A clear correlation can be observed between the particle yield and encapsulation efficiency at each of the nanocomposites, which means the higher the yield, the higher the drug entrapment that can be achieved, which resulted in similar drug contents in the nanomedicines: 4.81%, 4.76%, and 4.17% for doxorubicin and 4.35%, 5.03%, and 5.31% for sorafenib in the case of the Resomer RG 502H PLGA, the Resomer RG 752H PLGA, and PEG-PLGA copolymers, respectively.

### 3.3. In Vitro Sorafenib Release

In preliminary studies, we found that the release profile of the Resomer RG 752H PLGA was much more beneficial than that of the Resomer RG 502H PLGA because the Resomer RG 502H PLGA showed a high initial burst and then the release rate became very low. Thus, the nanocomposites of the Resomer RG 752H and the PEG-PLGA copolymers were investigated in biorelevant drug release studies. The released ratios of sorafenib and doxorubicin were determined by HPLC indirectly after the dissolution of washed nanocomposites was sampled at a predetermined time. The released amount was calculated from the remaining drug content in the nanoparticles.

**Figure 4:**
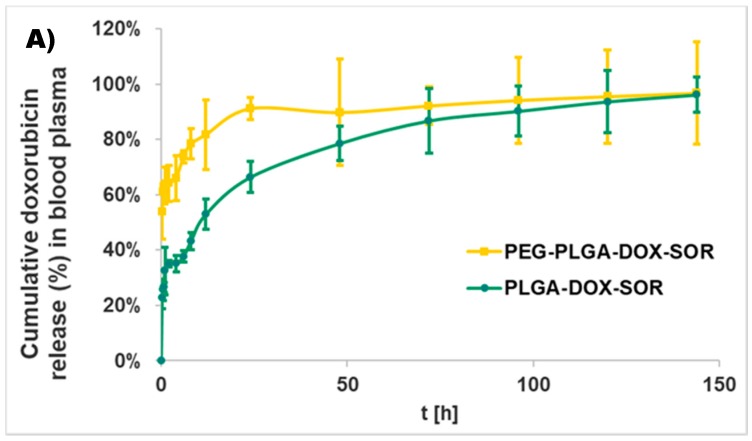

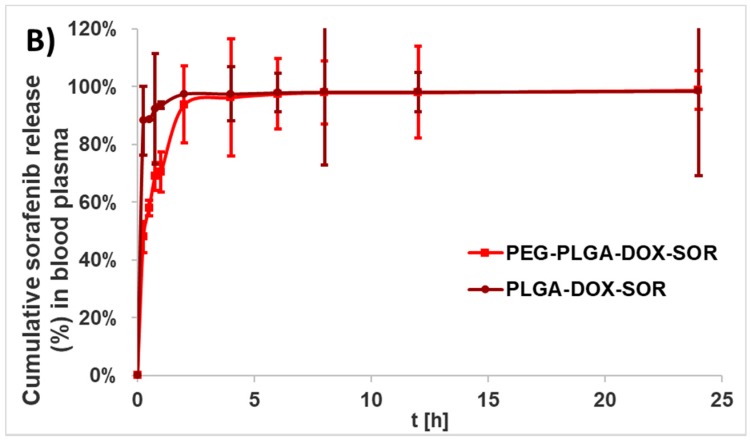
Doxorubicin (**A**) and sorafenib (**B**) release from PLGA-doxorubicin-sorafenib (PLGA-DOX-SFB) and PEG-PLGA-doxorubicin-sorafenib (PEG-PLGA-DOX-SFB) nanocomposites in human blood plasma. Data are presented as mean ± SD from three replicates for each concentration.

For the water-soluble drug doxorubicin HCl, the more hydrophilic carrier PEG-PLGA provided for a quicker release than PLGA with an initial burst of 54 ± 10%, while it was 23 ± 4% for PLGA-based nanoparticles (Figure 4A). After the burst release, both types of nanocomposites showed sustained release until the end of the study (1 week) and provided almost the complete liberation of doxorubicin (96 ± 6% for PLGA and 97 ± 19% for PEG-PLGA).

Sorafenib was released much more quickly from both of the carriers (Figure 4B). The initial burst of PLGA and PEG-PLGA was 88 ± 12% and 48 ± 5%, respectively.

The ratio of glycolide to lactide at different compositions enables control of the degree of crystallinity of the PLGA polymers. When the crystalline poly(glycolic acid) is co-polymerized with poly(lactic acid), the crystallinity degree decreases; consequently, the hydration rate and hydrolysis are enhanced. Thus, the degradation time of the copolymer is related to the ratio of monomers used in the synthesis. In general, the higher the content of glycolide, the quicker the rate of degradation [17]. In the Resomer RG 752H polymer, which was used as the PLGA matrix for the release studies, the lactide:glycolide ratio was 75:25; hence, a slower release was expected, especially for sorafenib. However, the extremely quick sorafenib release can be interpreted as the substantial influence of doxorubicin on sorafenib microencapsulation. It is hypothesized that, due to the doxorubicin incorporation, most of the sorafenib must be precipitated on the surface of the nanocomposites, which can be easily dissolved in the blood plasma due to the strong interaction of sorafenib and serum proteins [18].

**Figure 5:**
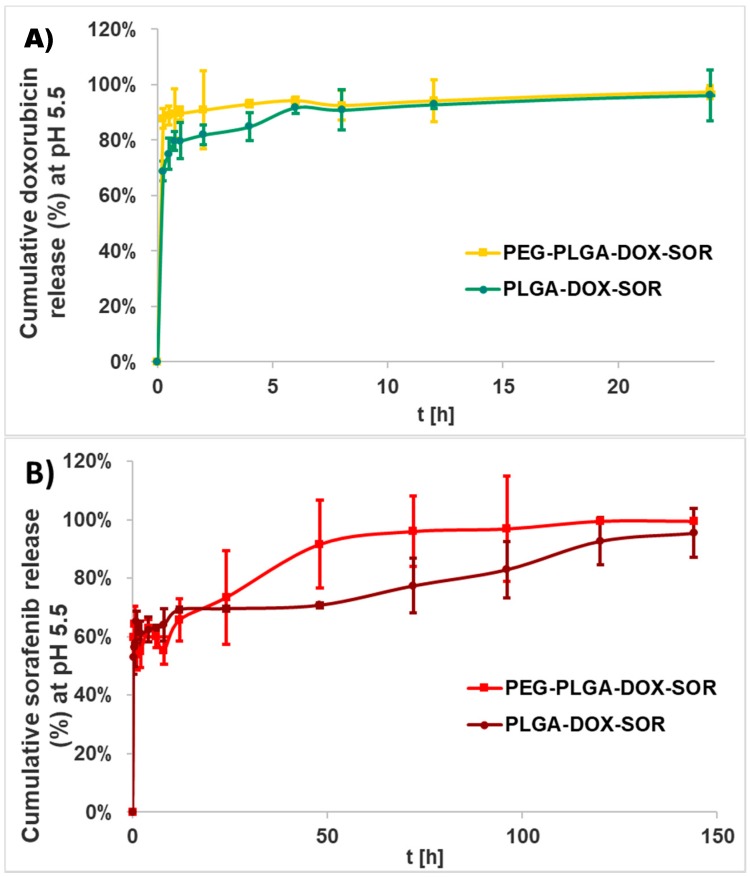
Doxorubicin (**A**) and sorafenib (**B**) release from PLGA-doxorubicin-sorafenib (PLGA-DOX-SFB) and PEG-PLGA-doxorubicin-sorafenib (PEG-PLGA-DOX-SFB) nanocomposites in ammonium acetate buffer (pH 5.5). Data are presented as mean ± SD from three replicates for each concentration.

The release of the active agents was investigated also under an acidic condition since the tumor microenvironment is generally acidic and nanoparticles accumulate generally in lysosomes that can be characterized by an internal acidic pH [19]. The release characteristics in acidic buffer were found to be opposite compared to those in human blood plasma; that is, doxorubicin was liberated within 1 day in both encapsulating polymers (Figure 5). Sorafenib release was completed in 6 days. The PEG-PLGA carrier displayed considerably faster release compared to the PLGA carrier, especially for sorafenib.

### 3.4. Cellular Uptake

Flow cytometry was used to study the in vitro cellular uptake by the HT-29 human cancer cell line. After 1 day of incubation with the Cyanine-5-conjugated nanocomposites, all of the living cells seemed to engulf nanoparticles according to the fluorescence activated cell sorting (FACS) experiments in each of the examined wells. There was a difference only among the amount of cells that was engulfed and expressed in different fluorescent intensity values (Table 3). A significantly higher amount of the nanocomposites prepared using PEG-PLGA was taken up by the cells than that using PLGA. It is also noted that the drug-containing nanoparticles were engulfed to a substantially higher extent than the blank nanoparticles, which might be the result of the changed surface characteristics.

### 3.5. Cytotoxicity

**Figure 6:**
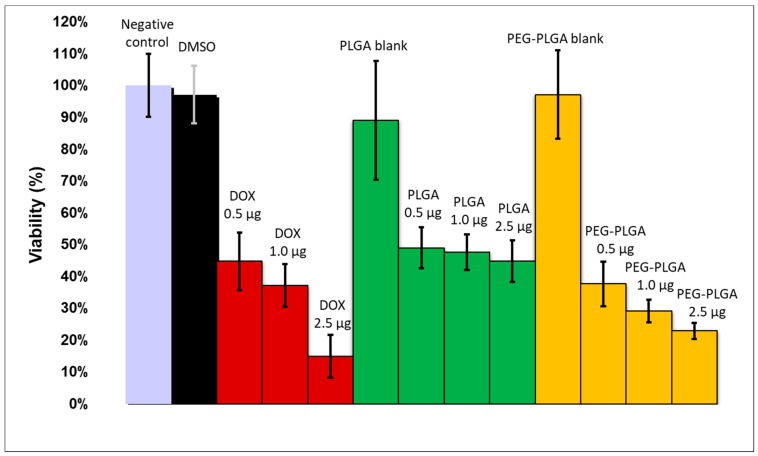
Viability of HT-29 cells by MTT assay due to different concentrations of doxorubicin (DOX) and sorafenib in solution (red columns), PLGA nanoparticles (green columns), and PEG-PLGA nanoparticles (yellow columns).

To determine the cytotoxic effect of active agents in solution and nanocomposites on HT-29 cancer cells, an MTT assay was performed. As shown in Figure 6, the cells remained viable in the negative control, DMSO-, and blank-nanoparticles-treated wells. The increasing concentration of drugs in solution decreased the cell viability to 15%. Drug-containing PLGA nanoparticles caused similar cytotoxicity at all the three concentration levels (viability: 49–45%). Drug-loaded PEG-PLGA nanocomposites reduced the cell viability more substantially (38–23%). The higher cytotoxicity of the PEG-PLGA nanoparticles compared to the PLGA nanocomposites is in accordance with their quicker drug release.

## 4. Conclusions

Doxorubicin and sorafenib co-loaded therapeutic nanocomposites were developed using PLGA- and PEG-PLGA-encapsulating polymers, respectively, by the double emulsion solvent evaporation method. The nanoparticles possessed promising physical and chemical properties; that is, a small size, high yield, high drug encapsulation efficiency, and high drug loading. The doxorubicin was released continuously within 6 days, while the sorafenib was released quickly during 24 h under biorelevant conditions. In an acidic tumor-simulated condition, the two agents presented opposing characteristics with accelerated doxorubicin and sustained sorafenib release. The hydrolysis of PEG-PLGA was quicker in both of the media. PEG-PLGA nanocomposites displayed higher cellular uptake, which harmonizes with the higher cytotoxicity experienced.

## Figures and Tables

**Table 1:** Mobile phase composition in HPLC analysis of doxorubicin (DOX) and sorafenib (SOR) co-loaded nanocomposites.

Time (min)	Methanol (%)	0.1% Tetrafluoroacetic Acid in H_2_O (%)
0.0	30.0	70.0
5.0	30.0	70.0
8.00	40.0	60.0
11.00	50.0	50.0
14.00	60.0	40.0
17.00	70.0	30.0
20.00	80.0	20.0
23.00	90.0	10.0
27.00	100.0	0.0
30.00	100.0	0.0
35.00	30.0	70.0

**Table 2:** Yield, size, polydispersity index (PDI), and encapsulation efficiency (EE) of doxorubicin (DOX) and sorafenib (SOR) co-loaded nanocomposites.

Polymer	PLGA RG 502H	PLGA RG 752H	PEG-PLGA
Yield (%)	70.0	49.7	75.3
Intensity mean diameter (nm)	164.6	142.2	177.2
PDI	0.203	0.123	0.076
EE (DOX) (%)	74	52	69
EE (SOR) (%)	67	55	88
DOX loading (%)	4.81	4.76	4.17
SOR loading (%)	4.35	5.03	5.31

**Table 3:** Fluorescent intensity values of blank as well as doxorubicin (DOX) and sorafenib (SOR) co-loaded PLGA and PEG-PLGA nanoparticles in HT-29 cellular uptake studies.

Sample	Negative Control	PLGA Blank	PLGA-DOX-SOR	PEG-PLGA Blank	PEG-PLGA-DOX-SOR
mean fluorescent intensity	22	17,105	22,243	21,846	45,765
SD (%)	4.5	1.7	30.0	3.9	24.8
